# Recent advances in pathophysiology of disseminated intravascular coagulation: the role of circulating histones and neutrophil extracellular traps

**DOI:** 10.12688/f1000research.12498.1

**Published:** 2017-12-18

**Authors:** Yasir Alhamdi, Cheng-Hock Toh

**Affiliations:** 1 Department of Clinical Infection, Microbiology and Immunology, Institute of Infection and Global Health, University of Liverpool, Liverpool, UK; 2 Roald Dahl Haemostasis & Thrombosis Centre, Royal Liverpool University Hospital, Liverpool, UK

**Keywords:** DIC, neutrophil extracellular, Thrombin

## Abstract

Disseminated intravascular coagulation (DIC) is an acquired condition that develops as a complication of systemic and sustained cell injury in conditions such as sepsis and trauma. It represents major dysregulation and increased thrombin generation *in vivo*. A poor understanding and recognition of the complex interactions in the coagulation, fibrinolytic, inflammatory, and innate immune pathways have resulted in continued poor management and high mortality rates in DIC. This review focuses attention on significant recent advances in our understanding of DIC pathophysiology. In particular, circulating histones and neutrophil extracellular traps fulfil established criteria in DIC pathogenesis. Both are damaging to the vasculature and highly relevant to the cross talk between coagulation and inflammation processes, which can culminate in adverse clinical outcomes. These molecules have a strong potential to be novel biomarkers and therapeutic targets in DIC, which is still considered synonymous with ‘death is coming’.

## Introduction

Disseminated intravascular coagulation (DIC) represents a major dysfunction in the hemostatic system, which is a physiological response to vascular injury. Upon injury, immediate interactions between components of the vessel wall and circulating blood lead to activation of the extrinsic and intrinsic pathways of coagulation to generate a burst of thrombin. Thrombin is immediately pro-coagulant in converting fibrinogen to fibrin but also mediates the anti-coagulant pathway by interacting with thrombomodulin (TM) to activate the protein C (PC) pathway. This controls the extent of localized clot formation, but when injury is of a systemic or sustained nature, regulation of thrombin generation is lost with adverse functional consequences. Owing to the ubiquitous nature of thrombin in affecting coagulation, fibrinolysis, and inflammation ^1– 3^, this can result in DIC and cause organ failure from microvascular thrombosis and endothelial barrier disruption.

This dynamic complexity in DIC pathogenesis is clinically important because its presence is well validated as an independent predictor of mortality ^4^. Development of DIC significantly increases risk of death beyond that of the underlying pathology. For example, DIC development in patients with sepsis and trauma doubles the risk of mortality ^5^. Despite this awareness, and the fact that its acronym could stand for ‘death is coming’, it remains poorly recognized by critical care clinicians and poorly managed because of the lack of high-quality evidence. DIC diagnosis is based on scoring a number of hemostatic parameters ^6^. Although measurements of the prothrombin time, fibrinogen, platelets, and fibrin-related products are generally available, changes in all of these parameters may not occur at the same time and can delay recognition and diagnosis. In some critical care settings, not all of these tests might be requested. Together with the lack of understanding that the manifestation of DIC can vary depending on primary disease-specific drivers of thrombin generation in causing multi-organ failure, this has resulted in poor management and mortality rates of 50% ^7^.

With the overall aim of improving our understanding of how DIC contributes to adverse clinical outcomes, this review will build upon key criteria in DIC. These were set out by the International Society on Thrombosis and Hemostasis (ISTH) Scientific and Standardization Sub-Committee (SSC) in describing how DIC can arise from the vasculature but also cause damage to the vasculature ^4^. Furthermore, the direct coupling of inflammation to coagulation processes facilitates the development of organ dysfunction. Specifically in this review, we will examine how extracellular histones and neutrophil extracellular traps (NETs) fit the DIC criteria and key principles in our understanding of DIC pathogenesis. Insight into this rapidly growing area of research could pave the way for improved clinician understanding and better approaches to manage the patient with DIC.

## Extracellular histones and neutrophil extracellular traps

While the intra-nuclear function of histones as proteins that package DNA into nucleosomes has been well understood ^7, 8^, it is their role when released extracellularly upon cellular damage or death that is of interest and relevance to DIC. The cytotoxicity of extracellular histones, whereby their neutralization in sepsis models with anti-histone antibodies or activated PC (APC) conveyed survival benefit were first described in 2009 ^9^. This discovery, followed by further *in vitro* and *in vivo* work, has translated into studies in patients with sepsis, trauma, and pancreatitis to illustrate the clinical relevance of extracellular histones and histone-DNA complexes (nucleosomes) to systemic inflammation, microvascular thrombosis ^10– 14^, organ injury ^10, 11, 15– 18^, and death. Extracellular histones may be found in the circulation (either free or complexed with DNA ‘nucleosomes’) or localized (and modified) as part of the extracellular traps released upon damage or activation of nucleated cells, primarily neutrophils. The various roles of neutrophils as key modulators of the complex interaction between innate immunity, inflammation, and coagulation (also known as ‘immunothrombosis’) are increasingly recognized and were recently well reviewed by Stiel *et al*. ^19^. The focus of this review is on one aspect of neutrophil contribution; which is mediation by NETs.

NETs were first described in 2004 as a neutrophil-derived amalgam of elastases, histones, and DNA that collectively trap and kill bacteria ^20^. Like histones, NETs have important physiological but potential pathological manifestations. Their uncontrolled or inappropriate release by neutrophils can contribute to the pathogenesis of sepsis, micro- and macro-vascular thrombosis, and multiple organ injury ^21– 26^. Consequences of NETs are typically site-specific, but breakdown products, such as cell-free DNA (cfDNA) or DNA-myeloperoxidase (DNA-MPO) complexes, can be found in the circulation ^27– 31^. However, cfDNA can arise from damaged cells and not only from NETs ^32^. As such, high circulating cfDNA levels should not be assumed to correlate directly with *in vivo* NET formation. Furthermore, intact NETs are structurally different and functionally dependent on its associated contents both locally and when cleaved and present in the circulation ^33, 34^.

**Figure 1.**
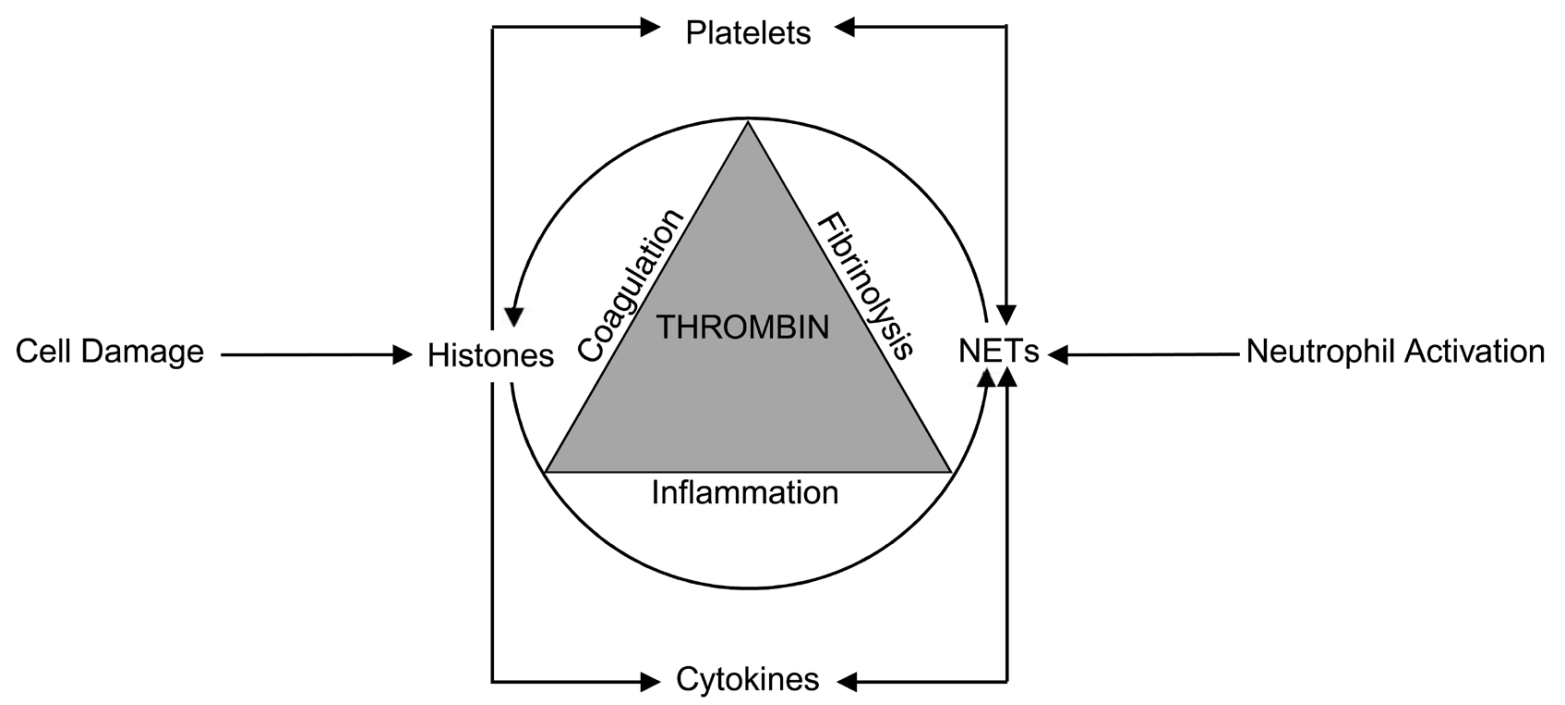
Bi-directional relationship between histones and neutrophil extracellular traps (NETs). Cell damage releases histones which trigger NET formation and the formed NETs are a source for both localized and systemic histone release. The increased thrombin generation, which is the hallmark of disseminated intravascular coagulation, simultaneously affects coagulation, fibrinolysis, and inflammation processes to amplify the reciprocal relationship between histones and NETs.

Importantly, there is a bi-directional relationship between NETs and histones ( Figure 1). First, NETs bear exposed histones (and numerous potent enzymes such as elastase) on their meshwork and therefore facilitate local histone-mediated cytotoxicity, pro-coagulant and pro-inflammatory effects. Histones can also be released from NETs into the circulation to disseminate its adverse effects ^35, 36^. Second, histones can directly stimulate neutrophils to form NETs ^10, 37, 38^ and therefore there is a vicious circle triggered by cellular injury that is then propagated by this bi-directional relationship between histones and NETs to promote further thrombin generation and contribute to DIC pathogenesis. These will be specifically detailed below in addressing how the various pathophysiological aspects of DIC can be contributed to by circulating histones and NETs.

## Relevance of histones and neutrophil extracellular traps to disseminated intravascular coagulation

The discussion will be divided into how histones and NETs contribute to the initiation, amplification, and propagation of coagulation activation ( Table 1).

**Table 1.** Mechanisms by which histones and neutrophil extracellular traps cause disseminated intravascular coagulation.

**Triggering coagulation** • Tissue factor expression on endothelial cells and macrophages (mediated by Toll-like receptors and pro-inflammatory pathways: nuclear factor-kappa B and activator protein 1) ^12, [Bibr ref-39]^ • Neutrophil extracellular traps bearing tissue factor ^40– 42^ • Pro-inflammatory cytokine release, production, and activation ^10, 43– 48^ • Cellular activation and injury, including platelets ^9, 10, 13, 26, 49– 54^
**Amplifying coagulation** • Reduced endogenous anti-coagulant activity by consumption, liver damage, and extravascular extravasation ^12, 16, 55– 59^ • Intrinsic pathway activation ^60– 63^ • Impaired fibrinolysis ^64– 66^
**Propagating coagulation** • Cytotoxic effects in circulation ^9, 10, 15, 49, 59, 67– 69^ • Circulating microparticle effects ^70– 73^ • Distant organ injury ^9, 10, 15, 74^

### Factors that trigger coagulation in disseminated intravascular coagulation

***Tissue factor expression.*** It is widely acceptable that the most important trigger of coagulation in sepsis and trauma-associated DIC is excessive tissue factor (TF) expression by circulating monocytes and its exposure from the vascular sub-endothelium following injury ^75^. This is supported by the observation that patients with DIC have significantly higher levels of circulating TF compared with controls ^76^. Exaggerated TF expression in septic and trauma DIC was historically attributed to systemic inflammation triggered by the invading microorganisms or their toxins (such as lipopolysaccharides) ^39– 41^. However, two recent studies have shown that extracellular histones can directly induce TF expression in a dose- and time-dependent manner on the surface of endothelial cells and macrophages via Toll-like receptor-4 (TLR-4) and TLR-2 and activation of the nuclear factor-kappa B (NF-κB) and activator protein 1 (AP-1) pathways ^12, 42^. With regard to NETs, recent studies have reported that NETs bear TF and contribute to thrombosis in myocardial infarction ^77^ and anti-neutrophil cytoplasmic antibody-associated vasculitis ^78, 79^. One study in the cancer setting reported significant interactions and correlations between NET components (primarily circulating DNA and nucleosomes) and TF-bearing microparticles culminating in overt DIC ^43^. Interestingly, both TF-bearing NETs and TF-bearing microparticles were found to be triggered by complement C5a ^79^, highlighting a significant interaction between the coagulant and innate immune systems that include complement in contributing to pathology.

***Pro-inflammatory cytokine involvement.*** In sepsis and trauma, the DIC process is directly linked into, and triggered by, the systemic inflammatory host response, of which pro-inflammatory cytokines play critical roles beyond induction of TF expression ^44^. Excessive cytokine activities disrupt the fine balance and cross talk between coagulant, anti-coagulant, and inflammatory pathways to augment the pro-coagulant phenotype ^44, 45^. Histones can directly induce the release of several pro-inflammatory cytokines, including interleukin-6 (IL-6), IL-1β, and tumor necrosis factor-alpha (TNF-α) ^10, 46– 48^. NETs act as scaffolds to potentiate IL production and activation ^80– 82^. Conversely, cytokines can induce NETosis ^83^ in a bi-directional relationship akin to that between histones and NETs ( Figure 1).

***Platelet activation.*** Platelets are important for both the initial burst and further sustenance of thrombin generation by acting as scaffolds on which further coagulation activation takes place ^84^ and through platelet-derived polyphosphate activation of factor XI ^85^. Platelets also promote a pro-coagulant phenotype via P-selectin expression which enables adherence to the vascular endothelium and leukocytes while also augmenting TF expression ^49^ and phosphatidylserine exposure on monocytes ^50, 51^ which collectively enhances thrombin generation ^51^. Histones can directly induce platelet activation via calcium influx with subsequent platelet aggregation and consumption *in vitro* and *in vivo* ^13, 52, 86^. Histones can also promote thrombin generation in a platelet-dependent manner via P-selectin expression, phosphatidylserine exposure, and FV/Va availability on platelet surfaces ^87^. Histone-induced TF expression and subsequent thrombin generation can further activate platelets. In terms of clinical relevance, a case control study in intensive care patients recently illustrated a strong association between high histone levels and subsequent platelet consumption and thrombocytopenia to translate the findings of histone-induced thrombocytopenia in animal models ^88^. In parallel, NETs can interact with platelets to induce platelet aggregation, polyphosphate release, and subsequent thrombin generation to cause intravascular coagulation in septic mice ^26^. Conversely, platelets can directly induce NETosis ^33, 89, 90^ ( Figure 1) and contribute to pathology, including that of transfusion-associated acute lung injury ^33, 90^.

***Vascular endothelial injury.*** The vascular endothelium, one of the biggest organs in the body, has a natural anti-coagulant surface mediated by the generation of APC ^91^, tissue factor pathway inhibitor (TFPI) ^92^, and expression of heparan sulfate and glycosaminoglycans that convey anti-thrombin (AT) activity ^93^. Endothelial cells also participate in fibrinolysis through release of tissue plasminogen activator (tPA) upon activation ^94, 95^. Therefore, damage or dysfunction of the vascular endothelium is another vital aspect of DIC pathogenesis. To this end, histone-induced toxicity on the vascular endothelium is well documented ^9^ and further extended in clinical studies with strong correlations observed between levels of circulating histones and soluble TM, a marker of endothelial cell injury, in critically ill patients ^10^. In addition, histones have been reported to induce the release of ultra-large von Willebrand factor (vWF) multimers ^96^, which are involved in platelet adhesion, platelet consumption, and microvascular thrombosis. Reports in patients with sepsis have indeed documented elevated levels of ultra-large multimers of vWF together with deficiency of a disintegrin and metalloproteinase with a thrombospondin type 1 motif, member 13 (ADAMTS-13), which is responsible for degradation of these multimers ^53, 54^, but the direct effect of histones on ADAMTS-13 is not currently known. As to NETs, a recent study showed that vascular endothelial cells have limited phagocytic capacity for NETs, which could result in poor NET clearance and subsequent damage to endothelial tight junctions to increase vascular permeability ^97^. Another study illustrated that matrix metalloproteinases within NETs contribute to endothelial dysfunction ^67^. Indeed, activated endothelial cells can also induce NETosis ^98^. As such, these histone- and NET-induced changes are also likely to play relevant roles in DIC pathogenesis.

A physical consequence of cellular injury induced by histones and NETs is the exposure of negatively charged phospholipid surfaces from membrane disruption ^99^. Availability of such phospholipid surfaces is highly pro-coagulant and can accelerate the prothrombinase reaction by 250,000-fold ^100^. In addition, histones can directly bind prothrombin and cause its auto-activation into thrombin. The implications would be that histones can directly generate thrombin without requiring coagulation activation ^101^. Although this reaction takes about eight hours and therefore may not be physiologically relevant, the available evidence points to the diversity in how histones directly contribute to thrombin generation and disseminate coagulation activation.

### Factors that amplify coagulation in disseminated intravascular coagulation

***Reduced endogenous anti-coagulant activity.*** The three endogenous anti-coagulant pathways—AT, PC, and TFPI—play a critical role in regulating the extent of clot generation. All of these pathways are significantly compromised in DIC. Declining trends in AT and PC levels can be used to identify patients in a non-overt stage of DIC before full decompensation ^6^. Low AT and PC levels have been validated as strong predictors of mortality in patients with sepsis and DIC ^55, 102^.

Although low levels of endogenous anti-coagulants can be due to pathological consumption by the excessive coagulopathy in DIC, other mechanisms also contribute to reduced activity and levels ^103^. Related to histones and NETs are two studies showing that histones can disrupt the PC pathway by downregulating TM ^12^ or dampening TM-dependent PC activation ^56^. This would impair APC anti-coagulant, anti-inflammatory, and cyto-protective functions ^57^, including its ability to proteolytically cleave histones ^9^. NET-associated elastases can potently degrade AT and TFPI ^58– 60^. In addition, two other potential mechanisms for endogenous anti-coagulant loss are reduced synthesis by the liver and loss to the extravascular space from enhanced vascular permeability ^60^. Histones can potentially contribute to both of these mechanisms by inducing liver injury and inflammation ^16, 61^ and significantly increasing vascular permeability through endothelial damage ^9, 10^.

***Intrinsic pathway activation.*** In physiological terms, the intrinsic pathway of coagulation plays an important role in increasing thrombin generation and accelerating hemostatic clot formation. This is well exemplified by the significant bleeding issues in patients without factor VIII or IX. cfDNA- and NET-bound DNA can exert pro-coagulant effects through activating the intrinsic pathway of coagulation via FXI and FXII ^62, 63^. Likewise, histones can activate the intrinsic pathway through an FXII-dependent mechanism, and histone-DNA complexes significantly contribute to elevated FXII in patients with overt DIC ^104^. Indirectly, histone-induced release of platelet polyphosphate can stimulate factor XI auto-activation as well as accelerate its thrombin-mediated activation ^105^.

***Impaired fibrinolysis.*** Impaired or excessive fibrinolysis is an important aspect of sepsis- and trauma-induced DIC, respectively ^64,65, 106^. All studies investigating the effects of histones, cfDNA, and NETs on the fibrinolytic system have consistently shown an overwhelmingly anti-fibrinolytic effect ^66, 68, 107^. This effect is mediated by enhanced clot resistance to fibrinolysis by plasmin and downregulation of plasminogen activation by tPA ^66, 68, 107^. As such, it appears that the effects of histones and NETs on the fibrinolytic system are relevant for sepsis-induced DIC but may not directly account for the hyper-fibrinolytic phenotype in trauma-associated DIC, although high histone levels in such patients may increase tPA release through significant endothelial stimulation and damage ^69^.

### Factors that propagate coagulation in disseminated intravascular coagulation

The development of multiple organ injury further augments thrombin generation and dysfunction in DIC. Microvascular thrombosis can be triggered by the factors discussed above in leading to organ ischemia and failure. There is increasing understanding of the role mediated by circulating histones in particular. In addition to causing direct injury to endothelial and other hematopoietic cells ^9, 10, 52, 99^, histones have been shown to mediate distant organ injury and dysfunction. In mice models of trauma ^10^ and sepsis ^15^, histones are major mediators of lung and cardiac injury and dysfunction, respectively. The clinical relevance of these findings has also been demonstrated in cohorts of critically ill patients with trauma ^10^ and sepsis ^15^. The evidence for the distant organ-damaging properties of histones in trauma comes from the consequent development of acute lung injury after significant non-thoracic trauma. Translational relevance is supported by the increased development of acute lung injury in patients with severe non-thoracic trauma with high histone levels. Similarly, sepsis patients without pre-existing cardiac disease were significantly more likely to develop new-onset cardiac arrhythmia (nine-fold increase) and left ventricular dysfunction (two-fold increase) if they had high histone levels ^15^. Equally, circulating histones can mediate renal ^108^, liver ^61, 109^, and brain ^109^ injury. Notably, the incubation of plasma or serum from critically ill patients (with sepsis, trauma, or pancreatitis) with cultured endothelial cells or cardiomyocytes induces cell death, which can be prevented in the presence of an antibody to histones ^10, 15^.

Cellular damage is associated with microparticle formation and release ^70, 71^. Their pro-coagulant properties include TF expression ^43, 78, 79^, as discussed above. Circulating microparticles from damaged or activated hematopoietic cells also have exposed phosphatidylserine and these surfaces provide attachment sites for coagulation factors, which contribute to thrombotic complications in inflammatory disorders ^72^. Significantly high levels of microparticles from activated endothelial cells and neutrophils were recently demonstrated in septic shock-induced DIC patients in whom elevated levels of NET surrogate markers (for example, nucleosomes and circulating DNA-MPO complexes) were evident ^73^. These microparticles may have synergistic pro-coagulant effects with NETs ^72^ and prime neutrophils to undergo NETosis by facilitating a pro-inflammatory environment, including the release of pro-inflammatory cytokines ^74, 110^.

These histone-induced cytotoxic effects not only are a manifestation of micro- and macro-vascular thrombosis due to the pro-coagulant effects described above but also are from direct ** cytotoxicity mediated by histone binding to cellular membranes with consequent pore formation, calcium influx, and overload ^10, 11, 52, 99, 111^. Fattahi *et al*. have demonstrated that after histone infusion into mice, histones localize (in order of concentration) in the lungs, spleen, kidneys, plasma, liver, heart, and brain ^112^. As histones unravel from DNA binding as part of nucleosomes, their cytotoxicity becomes apparent because of their ability to bind cell membrane phospholipids. However, in intact nucleosomes, where histone binding sites are covered by DNA, no cytotoxicity could be elicited ^86^. Collectively, these data suggest that circulating histones in patients with sepsis- or trauma-associated DIC are major mediators of distant organ injury and adverse clinical outcome.

## Summary and insights for the future

**Figure 2.**
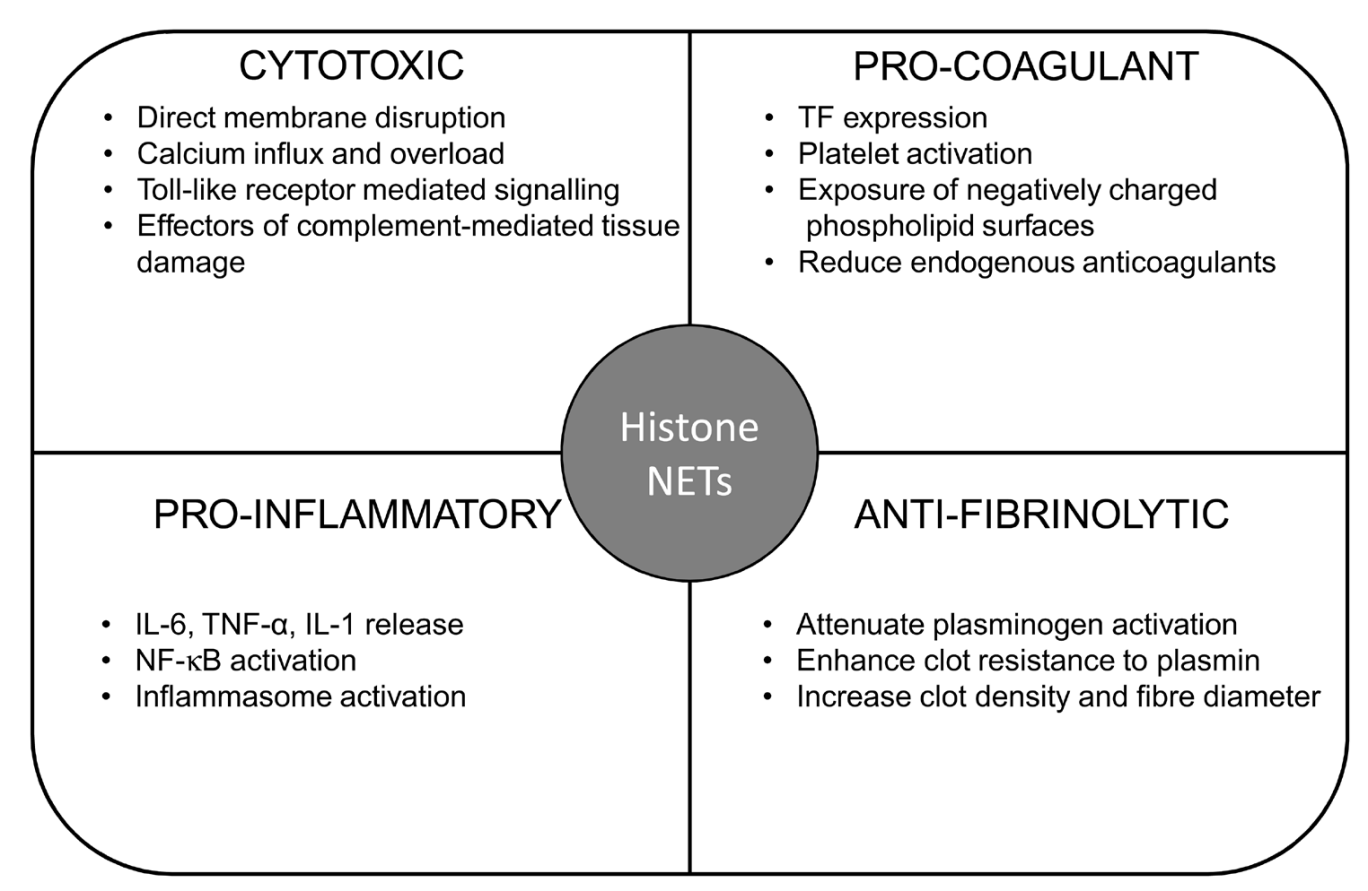
Functional consequences of circulating histones and neutrophil extracellular traps (NETs). Summary of pro-coagulant, anti-fibrinolytic, pro-inflammatory, and cytotoxic effects of histones and NETs. IL, interleukin; NF-κB, nuclear factor-kappa B; TF, tissue factor; TNF-α, tumor necrosis factor-alpha.

In this review, we have highlighted how extracellular histones and NETs fulfil the most important principles of DIC pathophysiology, as established by the ISTH SSC ^4^. First, histones can arise from endothelial cells following damage or from an exaggerated inflammatory response and in turn can mediate further significant damage to vascular endothelial cells. Directly and indirectly, histones can cause pro-inflammatory cytokine release and contribute to ‘inflammation gone amok’, as described in the ISTH communication ^4^. With the bi-directional relationship between histones and NETs, along with their functional consequences ( Figure 2), histones can be considered mediators of distant organ injury with NETs being the effectors of multi-organ failure.

As to how these findings can translate into better recognition of DIC, there are a number of studies showing histone-DNA complexes as important prognosticators in patients with DIC ^113^, and their levels correlate with increasing DIC scores ^114^. As such, there appears to be potential in using these molecules as biomarkers in early DIC before full decompensation occurs. This could be a major advancement in the diagnosis and management of critically ill patients at risk of DIC. One reason for this is that the current recommendations rely on a panel of coagulation tests collectively forming the ‘DIC score’, which signifies that the phenomenon is already under way or indeed advanced (overt DIC) rather than presents a target for early therapeutic intervention. Furthermore, the scoring system can vary between different societies and countries (for example, ISTH score, the Japanese Association for Acute Medicine Criteria, and the Japanese Ministry of Health and Welfare score), and this impacts considerably on specificities/sensitivities of detection, stage of DIC (for example, overt or non-overt) identification, and prognostic values ^90^. Therefore, there is an area of unmet need for biomarkers that can better improve standardization in diagnosing DIC.

One difficulty facing the implementation of such biomarkers is that there is no simple, rapid test for quantifying histones that could be suitable for the acute hospital setting. Furthermore, there is controversy regarding the level of circulating histones; some papers quote levels in the microgram-per-milliliter range using Western blotting quantification ^10, 15, 88^, whereas others suggest that levels are in the nanogram-per-milliliter or picogram-per-milliliter range using enzyme-linked immunosorbent assay (ELISA) ^13, 115, 116^. From our experience, current ELISAs are not sufficiently specific for histone measurement in clinical samples and this is due to interference from other plasma proteins. The same issue applies to NET measurement in patient samples. Currently, most studies rely on measuring cfDNA, histone-DNA, and DNA-MPO complexes as surrogate markers of NET formation ^27– 31^. Although these assays are a good development, they are also associated with problems relating to specificity, especially when the cfDNA may be released from other dying cells and not necessarily from NETs. Recent studies have illustrated promising potential for the use of neutrophil side fluorescence as a marker of neutrophil chromatin decondensation (hence NETosis) in predicting DIC development in patients with septic shock ^117^. This new marker correlated significantly (yet with a weak correlation coefficient) with circulating nucleosomes and DNA-MPO complexes in patients with DIC ^73^. Standardization for histone and NET measurements using accurate high output techniques is therefore a pressing need.

Nonetheless, these are exciting challenges to overcome. There is the potential for novel therapeutic approaches using modalities that neutralize histones (APC ^9^, anti-histone antibodies ^10, 11, 15^, recombinant TM ^13^, and heparin ^118^) or NETs (DNase ^20^, ADAMTS-13 ^119^, and PAD4-targeted therapy ^34^) or both. Many of these interventions are clearly anti-coagulant and could convey bleeding risk in patients with DIC if not used with caution. These would require well-designed randomized control trials using appropriate DIC patient populations (for example, with high circulating histones or NETs or both) as well as the potential for modified non-anti-coagulant versions as is the case with non-anti-coagulant heparins, which can neutralize circulating histones ^118^.

## Abbreviations

ADAMTS-13, disintegrin and metalloproteinase with a thrombospondin type 1 motif, member 13; APC, activated protein C; AT, anti-thrombin; cfDNA, cell-free DNA; DIC, disseminated intravascular coagulation; ELISA, enzyme-linked immunosorbent assay; IL, interleukin; ISTH, International Society on Thrombosis and Hemostasis; MPO, myeloperoxidase; NET, neutrophil extracellular trap; PC, protein C; SSC, Scientific and Standardization Sub-Committee; TF, tissue factor; TFPI, tissue factor pathway inhibitor; TLR, Toll-like receptor; TM, thrombomodulin; tPA, tissue plasminogen activator; vWF, von Willebrand factor.
